# Serum from Fibromyalgia Patients Activates Satellite Glial Cells in Mouse Peripheral Ganglia

**DOI:** 10.3390/cells15110974

**Published:** 2026-05-25

**Authors:** Menachem Hanani, Rachel Feldman-Goriachnik, Suhail Aamar

**Affiliations:** 1 Laboratory of Experimental Surgery, Hadassah-Hebrew University Medical Center, Mount Scopus, Jerusalem 91240, Israel; 2 Faculty of Medicine, Hebrew University of Jerusalem, Jerusalem 91120, Israel; 3 Rheumatology Unit, Assuta-Ashdod University Hospital, Ashdod 7747629, Israel; suhail_aamar@hotmail.com; 4 Faculty of Health Sciences, Ben-Gurion University of the Negev, Beer-Sheva 8410501, Israel; 5 Rheumatology Clinic, Hadassah-Hebrew University Medical Center, Mount Scopus Hospital, Jerusalem 91240, Israel

**Keywords:** fibromyalgia, sensory ganglia, sympathetic ganglia, satellite glial cells, glial activation

## Abstract

**Highlights:**

- We found that serum from patients with fibromyalgia activated satellite glial cells in mouse dorsal root, trigeminal and nodose ganglia, as well as the sympathetic superior cervical ganglia.
- These results suggest that serum factors can activate a variety of sensory and autonomic pathways, which may contribute to the symptoms in this disease.

**Abstract:**

Fibromyalgia (FM) is a complex syndrome associated with chronic widespread pain and with various other symptoms, including sleep and mood disturbances. Its underlying causes are not fully understood, and the lack of diagnostic blood tests and imaging, along with the absence of definitive treatments, makes management challenging. Recent studies showed that passive transfer of immunoglobulins from FM patients into mice activated satellite glial cells (SGCs) in mouse dorsal root ganglia (DRG), leading to pain behaviors. Here, we aimed to determine whether whole serum from FM patients activates mouse SGCs in DRGs and other ganglia that may be involved in FM’s diverse symptoms. Serum from FM patients (N = 15) and healthy controls (HCs, N = 8) was collected. Sera were incubated with different types of mouse sensory ganglia: DRG, trigeminal ganglion (TG), the nodose ganglion (NG), and the superior cervical sympathetic ganglion (Sup-CG). SGC activation was assessed by immunostaining of SGCs for the glial activation marker glial fibrillary acidic protein (GFAP). All the ganglia tested, DRG, TG, NG, and Sup-CG, displayed induced upregulation of GFAP labeling in SGCs after incubation with FM serum compared with HCs, indicating SGC activation by the serum. Similar responses were observed in both male and female mice. We conclude that serum from FM patients contains factors that can activate SGCs across various types of mouse ganglia, which may reflect the diverse symptom profile of FM. These findings provide evidence for pathogenic factors that could serve as a foundation for a diagnostic method for FM and require further purification and identification, hopefully paving the way for future targeted FM therapy.

## 1. Introduction

Fibromyalgia (FM) is a chronic pain syndrome accompanied by multiple symptoms [1]. Its prevalence is 3–5% in the Western population, mainly affecting females [1,2,3], and its etiology remains unclear. FM diagnosis is based on the presence of a combination of diffuse and chronic widespread pain, along with four categories of disturbance: non-restorative sleep, fatigue, cognitive or memory deficits, and various physical syndromes without demonstrable structural or organic pathology. The name of this syndrome has changed over the years; it has been called “fibrositis” to suggest inflammation, “myofascial pain syndrome” to highlight the muscles and fascia as the main involved structures, “pain amplification syndrome”, indicating sensitivity to minor triggers, and “central sensitization syndrome” [3,4,5,6,7]. The latter implies that peripheral sensory inputs (e.g., tactile, thermal) provoke abnormal responses in the central nervous system (CNS) [7]. The nociplastic pain in FM is related to hyperactivity in brain regions that process pain signals, along with decreased activity of inhibitory pathways [6]. Additionally, there is evidence of increased sympathetic activity in FM [8,9]. FM is often described as “mysterious” and is surrounded by controversy; even its very existence as a syndrome has been questioned by some authors [10,11].

The diagnosis of FM is a major obstacle in patient care due to the numerous associated comorbidities and the absence of biomarkers [10,11,12,13,14]. There is no generally accepted treatment for FM, which is explained by the lack of consensus on its diagnosis and, especially, its etiology.

Most investigators attribute the pain in FM to central sensitization [7,10], but there is also evidence for a peripheral contribution. Evidence in support of for this idea is the loss of small nerve fibers in the skin in 50% of FM patients, which is associated with greater severity and more pronounced CNS changes [15,16,17]. Also, abnormal spontaneous electrical activity was recorded in nociceptive fibers in FM patients [18]. Further evidence for peripheral involvement in FM is that neutrophil-derived mediators sensitized peripheral nerves [19]. A recent publication [20] showed that the intestinal microbiome contributes to FM through peripheral mechanisms.

Sensory ganglia are crucial for transmitting pain signals from the periphery to the CNS, and recent data suggest a role for them in FM [21]. The main sensory ganglia are DRGs, which innervate most body regions, including internal organs, the trigeminal ganglia (TG), which innervate the face, teeth, and part of the scalp, and the nodose ganglia (NG), which innervate internal organs, such as the lungs, heart, and bowel. Abnormal neuronal activity in these ganglia is a major factor in chronic pain [22,23]. Neurons in sensory ganglia are surrounded by satellite glial cells (SGCs), which form functional neuron–SGC units. Animal studies showed that SGCs play an important role in the generation and maintenance of chronic pain [24,25]. Evidence for the possible role of SGCs in FM was obtained in experiments in which immunoglobulins (IgGs) were transferred from FM patients into mice, which induced pain behavior in the mice [21,26]. Moreover, the FM IgGs were found to bind to SGCs in the DRGs of both humans and mice and to activate them, as evidenced by glial fibrillary acidic protein (GFAP) upregulation [21]. The severity of FM was correlated with serum levels of IgGs [26]. These studies indicate that, in humans, as in rodents, SGCs might contribute to chronic pain. These studies, combined with those mentioned in the paragraph above, lend considerable support to the idea that the peripheral nervous system plays a role in FM pain.

In addition to the pain pathways, the autonomic nervous system, and in particular the sympathetic nervous system (SNS), is also relevant to FM. There is evidence for SNS overactivity in FM [27], which may be related to some FM symptoms. Resting sympathetic activity is higher in FM compared with HCs [9]. Also, in FM patients heart rate variations are reduced [28] and baroreflex sensitivity is lower compared with HCs [29]. This seemingly paradoxical behavior—sympathetic hyperactivity with hyporeactivity—has been noted [28], and was explained by adrenergic receptor desensitization and downregulation induced by chronic activity [9].

Satellite glial cells are also present in sympathetic ganglia [25,30], and can influence neuronal activity [31]. There is no information on the involvement of SGCs of the SNS in FM. In this work, we studied SGCs in the mouse superior cervical ganglion (Sup-CG), which innervates the heart, neck, and face.

Recent studies on the role of SGCs in mediating chronic pain suggest a novel approach to understanding pain in FM. Currently, all available information on this topic is limited to the DRGs. In this work, we examined how SGCs in other peripheral ganglia, the TG, the NG, and the Sup-CG, are influenced by factors in the serum from FM patients.

## 2. Materials and Methods

### 2.1. Patient Selection

Participants were patients from the rheumatology clinic at Hadassah University Hospital in Jerusalem, diagnosed with FM after meeting the modified 2010/2011 American College of Rheumatology diagnostic criteria, and the 2016 revised criteria [32]. Diagnosis of FM was made when levels of the widespread pain index (WPI) and the symptom severity score (SSS) were sufficiently high (WPI ≥ 7 and SSS ≥ 5, or WPI 3–6 and SSS ≥ 9), with a minimum of 12 on the polysymptomatic distress (PSD) scale [33]. The WPI is a 0–19 count of painful non-articular body regions, and the SSS is a 0–12 measure of symptom severity that includes four aspects (scored 0–3): fatigue, sleep, somatic, and cognitive problems. The PSD scale, a 0–31 measure, was calculated by summing the WPI score and SSS for each patient [33]. PSD scores over 12, indicating FM, were categorized into 5-score groups: 12–16 as mild, 17–21 as moderate, 22–26 as severe, and 27–31 as very severe. Only FM patients with a severity score over 24 on the PSD scale were recruited for this study. Patients aged 18 years or older were included, in accordance with Helsinki Committee Approval (No. 0562-23HMO). Only primary FM patients were included. Patients with comorbidities such as rheumatic disease, painful orthopedic or neurological issues, or significant psychiatric conditions were excluded. Fifteen FM patients (14 females, one male) and eight healthy controls (HCs, all females) signed informed consent and were recruited for the study. A 10 mL blood sample was taken from each participant for serum separation, divided into 250 µL aliquots, and stored at −80 °C.

### 2.2. Immunohistochemistry

BALB/c mice aged 2–5 months old (males: females 1:1), weighing 19–23 g, were used. The procedures were approved by the Animal Care and Use Committee of the Hebrew University and conformed to the National Institutes of Health standards for the care and use of laboratory animals. Mice were sacrificed by CO_2_ inhalation, and ganglia (DRG L4,5; TG, NG, and Sup-CG) were removed from male and female mice and placed in ice-cold Krebs solution containing (mM): 120.9 NaCl, 5.9 KCl, 14.4 NaHCO_3_, 2.5 MgSO_4_, 2.5 CaCl_2_, 1.2 NaH_2_PO_4,_ and 11.5 glucose, at pH 7.4. Then, they were incubated for 2 h with serum diluted at a 1:4 ratio in Krebs solution in a CO_2_ incubator at 37 °C, and then fixed in 4% paraformaldehyde in 0.1 M phosphate buffer (pH 7.4) for 90 min at room temperature, washed in phosphate-buffered saline (PBS), and incubated overnight at 4 °C in PBS with 20% sucrose before freezing in Tissue-Tek embedding medium. We chose a 2 h incubation time because previous studies have shown that this duration is sufficient for inducing biochemical changes in cells of sensory ganglia [34,35]. Sections were cut 10 μm thick using a cryostat (Leica, Wetzlar, Germany) and thaw-mounted on glass slides, washed and incubated in a blocking solution containing 3% bovine serum albumin (BSA) in PBS with 0.3% Triton X-100 for 2 h at room temperature, and then incubated for 2 h with an antibody against GFAP (Dako, Glostrup, Denmark; 1:400 in PBS +1% BSA) overnight at 4 °C. For controls, the serum was omitted. Sections were washed in PBS and incubated with a secondary antibody, donkey anti-rabbit conjugated to Alexa Fluor 594 (Abcam, Cambridge, UK; 1:400), and 10 μM 4,6-diamidino-2-phenylindole dihydrochloride (DAPI) to stain the nuclei, for 2 h at room temperature. Finally, sections were washed, observed under a fluorescence microscope, and photographed with a digital camera. Microscope fields (315 × 235 μm) were selected randomly. All the images were taken under identical conditions and analyzed in a blinded manner. Neuronal profiles, containing the nuclei, which were surrounded by GFAP-positive SGCs by more than 50% of their circumference, were counted and expressed as a % of the total number of nucleated neuronal profiles in the field. This criterion was used because the SGC sheath can be partly invisible under light microscopy as it is very thin. Four fields from different non-adjacent sections were analyzed for each ganglion and then averaged.

### 2.3. Statistics

Data were analyzed using an unpaired two-tailed *t*-test. Values are expressed as mean ± SEM. *p* < 0.05 was considered statistically significant.

## 3. Results

**Figure 1:**
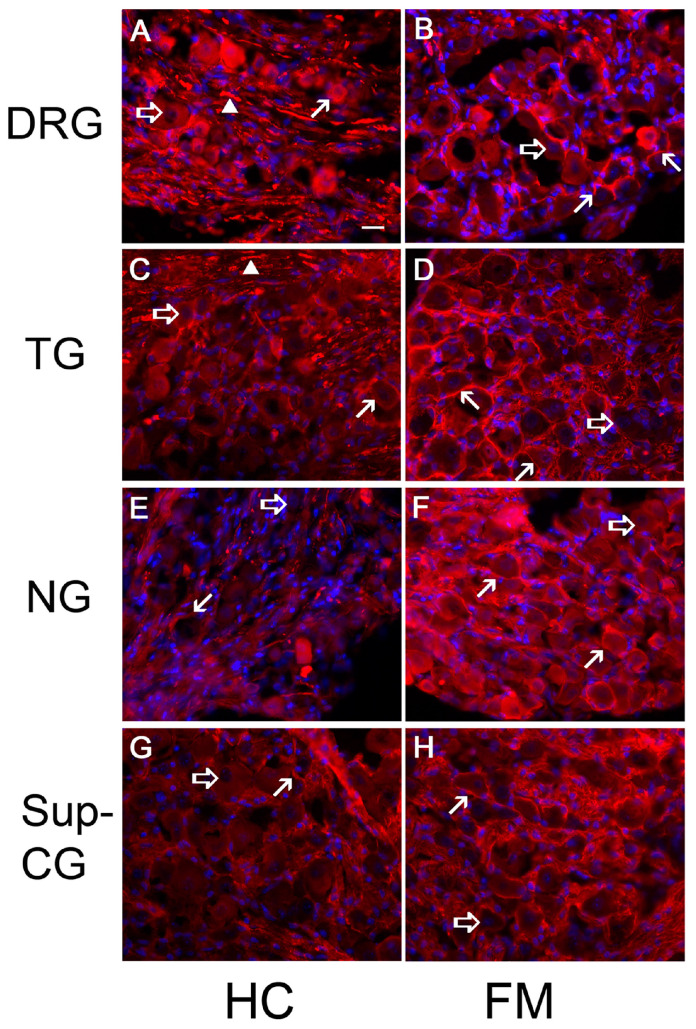
Sera from fibromyalgia (FM) patients activate satellite glial cells (SGCs) in mouse dorsal root ganglia (DRG), trigeminal ganglia (TG), nodose ganglia (NG), and superior cervical ganglia (Sup-CG). Left panel: (**A**,**C**,**E**,**G**) from healthy controls (HCs), and right panel: (**B**,**D**,**F**,**H**) from FM patients. Sections were stained for the activation marker glial fibrillary acidic protein (GFAP, red labeling). Labeled SGCs are seen as rings around the unstained neurons. Arrows indicate several GFAP-labeled SGCs. Cell nuclei were labeled with DAPI (blue); SGC nuclei are small and brightly stained with DAPI. Neuronal nuclei are larger and more faintly stained. Several neuronal nuclei are indicated with open arrows. Schwann cells are indicated with arrowheads. Scale bar for (**A**–**H**), 20 µm.

**Figure 2:**
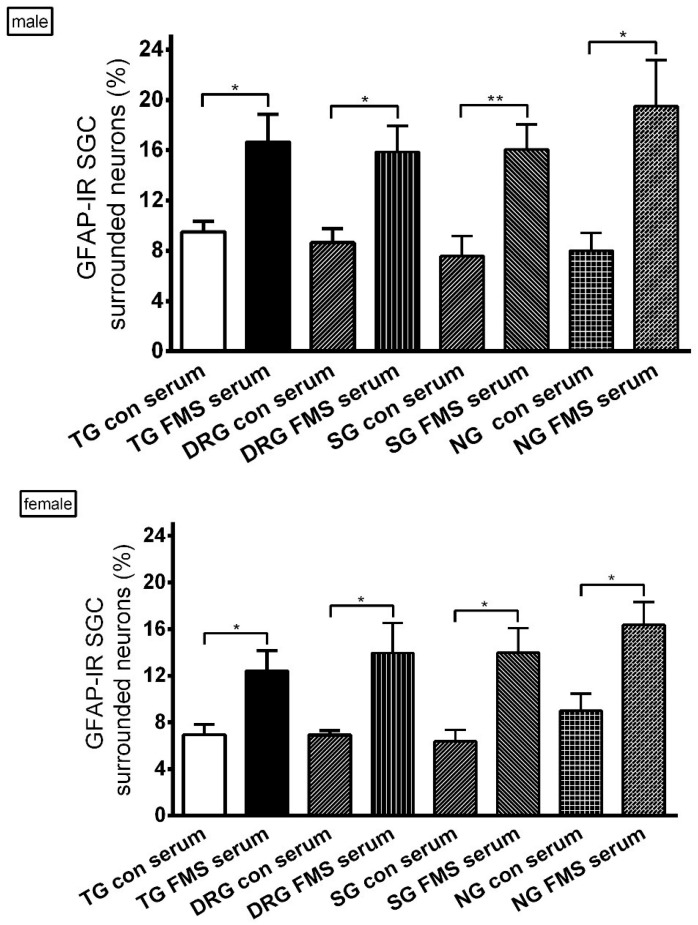
Quantitation of the GFAP immunostaining under FM and HC sera. Top, results for ganglia from male mice. Bottom, results for female mice. Error bars represent mean ± SEM. * indicates *p* < 0.05. ** indicates *p* < 0.01. The number of ganglia per bar was (from left to right) 4, 7, 4, 7, 6, 8, 4, 4 for male mice, and 7, 10, 6, 9, 4, 9, 4, 4 for female mice. Abbreviations: DRG, dorsal root ganglia; FMS fibromyalgia syndrome; HC, healthy control; NG, nodose ganglion; SG, superior cervical ganglion.

We first asked whether incubating intact DRGs with FM serum would activate SGCs, as previously observed in DRGs removed from mice injected with IgG from FM patients [21]. Freshly isolated DRGs were incubated with sera from FM patients and HCs diluted 1:4 in Krebs solution for 2 h, then fixed, sectioned, and immunostained for GFAP. GFAP immunostaining was mainly located in SGCs, which was verified with DAPI staining; SGC nuclei were small and stained intensely with DAPI, whereas neuronal nuclei were larger and faintly stained. As shown in Figure 1A,B and Figure 2, the percentage of neurons surrounded by a rim of GFAP-positive cells was approximately doubled in ganglia treated with FM serum compared with HC serum. The GFAP immunostaining results for incubation of the ganglia with HC serum were similar to those observed when the ganglia were incubated in Krebs solution [34]. A proportion of GFAP-immunopositive SGCs in controls was also reported by other authors [23]. Some neurons appear to be GFAP-immunopositive, apparently due to non-specific binding of antibodies. Some Schwann cells were GFAP-positive (Figure 1A,C), as was noted previously [36] There was no significant difference between the results obtained with DRGs from male and female mice (Figure 2). This result validated the method and indicated that serum might be a suitable tool for learning about FM pathophysiology, as found recently by other groups [37,38].

Next, we compared the DRG staining results with those from two other sensory ganglia, TG and NG, using the same experimental protocol. From Figure 1 and Figure 2, it seems clear that SGCs in these ganglia were activated by FM serum in a manner very similar to that of SGCs in DRG. Finally, we examined SGCs in a sympathetic ganglion, the Sup-CG, and again found that the FM serum upregulated GFAP in SGCs (Figure 1 and Figure 2). For all ganglion types, the results were similar for ganglia obtained from male and female mice (Figure 2). It can be concluded that for all four ganglion types, DRG, TG, NG, and Sup-CG, incubation in serum from FM patients induced an upregulation of labeling for GFAP.

## 4. Discussion

Reports on molecules in the serum of FM patients that induce pain behavior in mice, bind to SGCs in their DRGs, and activate them have attracted considerable attention because they provide a possible mechanistic explanation for pain in FM patients [21,26]. Still, this leaves open the question of whether SGCs in other types of peripheral ganglia can bind to, and be activated by, factors in the serum of FM patients. We show here that SGCs in two other types of mouse sensory ganglia (TG and NG), and also in a sympathetic ganglion (Sup-CG), are activated by FM serum. The results also show that serum from FM patients induces activation of mouse SGCs in vitro. These findings broaden the scope of potential humoral factors and targets that might contribute to the pathophysiology of FM.

We did not observe differences in the results for ganglia obtained from male and female mice across all four ganglion types. This correlates with the results of Goebel et al. [21], who found no difference in behavioral responses to IgG injections from FM patients between male and female mice. This suggests that the behavioral findings are unlikely to be due to increased sensitivity of SGCs to IgGs. (In the immunohistochemical results, the sex of mice was not specified.) Instead, it can be proposed that the activation of SGCs in FM patients (who are mostly females) may be due to the higher level of blood factors, such as IgGs, rather than a higher binding affinity of SGCs for these agents.

The current work and a previous study [21] showed that component(s) in FM serum activate SGCs in sensory ganglia. The mechanisms connecting this activation to pain in FM are not yet clear, but several ideas can be suggested. There is evidence that SGC activation leads to hyperexcitability of sensory neurons in DRG and TG [24]. This appears to occur by the release of chemical mediators, such as cytokines, from SGCs, which sensitize the sensory neurons. In addition, the release of ATP from SGCs can excite the neurons. These, and possibly other processes, induce neuronal firing, which results in augmented nociceptive inputs into the spinal cord.

In most accounts of FM, pain in somatic regions is emphasized, though pain in FM is “head to toe”. Severe pain syndromes involving the face and head are well known in the general population, and it is established that many FM patients experience pain mediated by the trigeminal system, such as temporomandibular pain and burning mouth syndrome [39,40]. Sensations from the head and face are transmitted via the trigeminal nerves, but there is no information on SGCs within the TG in FM. Here, we demonstrated that FM serum activates SGCs in mouse TG, as it does in the DRG, suggesting that head and face pain in FM patients may be linked to SGC activation, as has been proposed for somatic pain [21,41].

The NG contains the cell bodies of neurons that provide sensory innervation to many internal organs, and is essential for reflexes such as swallowing and coughing. It is also important for maintaining the inflammatory reflex, which is mediated by parasympathetic pathways [42]. Evidence suggests that in FM, there is an imbalance between sympathetic and parasympathetic activity, with low vagal tone and high sympathetic one compared with normal subjects [43]. We observed activation of SGCs in the NG after incubation with FM serum. If such activation increases the excitability of nodose ganglion neurons, it would lead to increased activity in vagal pathways and possibly the inflammatory reflex. The role of vagal afferents in pain is complex [43] and it is difficult to predict how the effects mentioned above would manifest in FM, but this topic warrants further study.

The autonomic nervous system (ANS) plays a key role in the symptoms of FM. There is evidence of overactivity of the sympathetic tone in FM, which may contribute to symptoms such as an abnormal heart rate [8,27]. Neurons in sympathetic ganglia release norepinephrine, which can cause pain [44]. The Sup-CG, one of the largest sympathetic ganglia, innervates the heart [45,46], pineal gland [47], and other organs. The pineal gland produces melatonin, a crucial hormone for the sleep–wake cycle, and therefore abnormal activity in Sup-CG might lead to sleep disturbances. Indeed, melatonin level is abnormally high in FM patients [48].

**Limitations**. In this study, we used sera from FM patients and compared their effects on peripheral ganglia with those of HC serum. This is unlike several previous studies in which IgGs isolated from the sera of FM patients or HCs were injected into mice, followed by DRG removal and GFAP staining. In addition to IgGs, FM serum contains many other bioactive molecules. Thus, we cannot specify the nature of the factor(s) that induced the observed effects in our experiments. However, recent studies have reported that, in addition to IgGs, other bioactive molecules are upregulated in FM serum, including the immunoregulatory proteins CD40 and CD40L [49]. Moreover, Seefried et al. [38] incubated DRG sections with FM serum and found protein binding to a variety of receptors (e.g., serotonin 5HT1A receptors) in both neurons and SGCs. Likewise, acute application of FM serum onto DRG neurons and SGCs in tissue culture evoked a physiological response in these cells [37]. Thus, using serum can reveal new aspects that might be missed by focusing on isolated IgGs. Future work is needed to identify the full spectrum of changes between HC and FM sera that underlie SGC activation, and possibly other relevant actions of FM serum.

## 5. Conclusions

Fibromyalgia is a polysymptomatic disorder with multiple manifestations affecting various systems. Our study shows that serum from FM patients activates mouse SGCs in various peripheral ganglia, including DRGs, via factors in the serum, which are believed to be related to FM’s diverse symptoms. Further research and validation of these results, as well as trials to purify these factors, are strongly warranted.

## Data Availability

The data that support the findings of this study are available from the corresponding authors upon reasonable request.

## Abbreviations

The following abbreviations are used in this manuscript:

ANS	Autonomic nervous system
CNS	Central nervous system
DAPI	4,6-diamidino-2-phenylindole dihydrochloride
DRG	Dorsal root ganglion
GFAP	Glial fibrillary acidic protein
FM	Fibromyalgia
HC	Healthy controls
IgG	Immunoglobulin G
NG	Nodose ganglion
PBS	Phosphate-buffered saline
PSD	Polysymptomatic distress
SGC	Satellite glial cell
Sup-CG	Superior cervical sympathetic ganglion
SNS	Sympathetic nervous system
